# γδT cells but not αβT cells contribute to sepsis-induced white matter injury and motor abnormalities in mice

**DOI:** 10.1186/s12974-017-1029-9

**Published:** 2017-12-20

**Authors:** Xiaoli Zhang, Eridan Rocha-Ferreira, Tao Li, Regina Vontell, Darakhshan Jabin, Sha Hua, Kai Zhou, Arshed Nazmi, Anna-Maj Albertsson, Kristina Sobotka, Joakim Ek, Claire Thornton, Henrik Hagberg, Carina Mallard, Jianmei W. Leavenworth, Changlian Zhu, Xiaoyang Wang

**Affiliations:** 1 Henan Key Laboratory of Child Brain Injury, The Third Affiliated Hospital of Zhengzhou University, Zhengzhou, 450052 China; 2 Perinatal Center, Department of Neuroscience and Physiology, Sahlgrenska Academy, University of Gothenburg, Box 432, 405 30 Gothenburg, Sweden; 3 Department of Obstetrics and Gynecology, Sahlgrenska Academy, University of Gothenburg, Gothenburg, Sweden; 4 Center for Brain Repair and Rehabilitation, Institute of Neuroscience and Physiology, Sahlgrenska Academy, University of Gothenburg, Box 436, 405 30 Gothenburg, Sweden; 5 Department of Perinatal Imaging and Health, Division of Imaging Sciences and Biomedical Engineering, King’s College London, King’s Health Partners, St. Thomas’ Hospital, London, SE1 7EH UK; 6 Department of Cardiovascular Medicine, Ruijin Hospital, Shanghai Jiao Tong University Medical School, Luwan Branch, Shanghai, China; 7 Department of Women’s and Children’s Health, Karolinska University Hospital, Karolinska Institute, Stockholm, Sweden; 8 Department of Neurosurgery, The University of Alabama at Birmingham, Birmingham, AL 35233 USA; 9 Department of Microbiology, The University of Alabama at Birmingham, Birmingham, AL 35233 USA; 10 Department of Pediatrics, The Third Affiliated Hospital of Zhengzhou University, Zhengzhou, China

**Keywords:** Sepsis, White matter injury, T lymphocytes, Behavior tests, Preterm

## Abstract

**Background:**

Infection and sepsis are associated with brain white matter injury in preterm infants and the subsequent development of cerebral palsy.

**Methods:**

In the present study, we used a neonatal mouse sepsis-induced white matter injury model to determine the contribution of different T cell subsets (αβT cells and γδT cells) to white matter injury and consequent behavioral changes. C57BL/6J wild-type (WT), T cell receptor (TCR) δ-deficient (*Tcrd* ^−/−^, lacking γδT cells), and TCRα-deficient (*Tcra* ^−/−^, lacking αβT cells) mice were administered with lipopolysaccharide (LPS) at postnatal day (PND) 2. Brain myelination was examined at PNDs 12, 26, and 60. Motor function and anxiety-like behavior were evaluated at PND 26 or 30 using DigiGait analysis and an elevated plus maze.

**Results:**

White matter development was normal in *Tcrd* ^−/−^ and *Tcrα* ^−/−^ compared to WT mice. LPS exposure induced reductions in white matter tissue volume in WT and *Tcrα* ^−/−^ mice, but not in the *Tcrd* ^−/−^ mice, compared with the saline-treated groups. Neither LPS administration nor the T cell deficiency affected anxiety behavior in these mice as determined with the elevated plus maze. DigiGait analysis revealed motor function deficiency after LPS-induced sepsis in both WT and *Tcrα* ^−/−^ mice, but no such effect was observed in *Tcrd* ^−/−^ mice.

**Conclusions:**

Our results suggest that γδT cells but not αβT cells contribute to sepsis-induced white matter injury and subsequent motor function abnormalities in early life. Modulating the activity of γδT cells in the early stages of preterm white matter injury might represent a novel therapeutic strategy for the treatment of perinatal brain injury.

**Electronic supplementary material:**

The online version of this article (10.1186/s12974-017-1029-9) contains supplementary material, which is available to authorized users.

## Background

Perinatal infection can induce a systemic inflammatory response characterized by the activation of various types of immune cells, including T lymphocytes, in infants, which increases the risk of cerebral white matter injury. Such injury is associated with neurological impairment and cerebral palsy, especially in preterm infants [1–5]. Neonates born at 24–32 weeks of gestation are particularly vulnerable to white matter injury, which is characterized by the death and/or developmental arrest of pre-oligodendrocytes [6, 7]. Rodents at postnatal days (PNDs) 2–5 have the highest percentage of pre-oligodendrocytes and thus mimic the vulnerable developmental stages of preterm human infants [8].

The endotoxin lipopolysaccharide (LPS) is an important component of the outer membrane of Gram-negative bacteria (e.g., *Escherichia coli*) and is a leading cause of bacterial sepsis in infants. LPS is widely used for inducing a systemic inflammatory response such as sepsis and cerebral white matter injury in newborn rodents [9–12]. LPS initiates downstream pathways to induce cytokine responses by binding to Toll-like receptor (TLR) 4, which is expressed on both immune and non-immune cells [13, 14]. The immune cells include dendritic cells (DCs) and T lymphocytes. While T cells are recognized as one of the important immune cell types that play a central role in fighting infection, even in early life [15], DCs act as antigen-presenting cells or accessory cells that initiate or amplify T cell activation. T cells are categorized into αβT cells and γδT cells according to the T cell receptors (TCRs) they express. LPS activates T cells either directly through interaction with TLR4 on T cells [13] or indirectly through stimulation of DCs that subsequently activate T cell responses [16–18]. Currently, little is known regarding how the LPS-induced crosstalk between the brain and the distinct T cell subsets in the immune system in early life impacts brain development and behavior at later ages.

Here, we investigated whether γδT cells and/or αβT cells contribute to sepsis-induced white matter injury in neonatal mice and whether depletion of these T cells can improve neurobehavioral performance in both the short and long terms under physiological and pathological conditions.

## Methods

### Animals

**Fig. 1:**
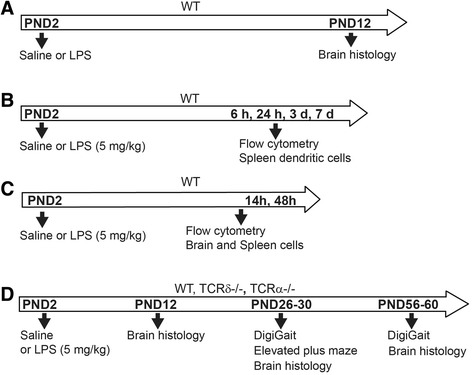
Schedule of experimental procedures. **a** Saline and LPS were administered subcutaneously in WT (C57BL/6J) mice, and white matter was evaluated at PND 12. **b** Spleen dendritic cells were counted at 6 h, 24 h, 3 days, and 7 days after LPS injection in WT mice. **c** γδT cells and dendritic cell activation in the spleen and brain were examined at 14 and 48 h after LPS injection in WT mice. **d** White matter evaluation and behavior tests were carried out at young (PNDs 26–30) and adult (PNDs 56–60) ages after LPS injection in WT, *Tcrd* ^−/−^, and *Tcrα* ^−/−^ mice

Wild-type (WT) C57BL/6J, T cell receptor δ-deficient (*Tcrd* ^−/−^, *B6.129P2-Tcrdtm1Mom/J*), and T cell receptor α-deficient (*Tcra* ^−/−^, *B6.129S2-Tcratm1Mom/J*) mice were obtained from the Jackson Laboratory and continuously bred in an accredited animal facility at the University of Gothenburg (Experimental Biomedicine, Sweden). The mice were housed with a 12-h light/dark cycle and had free access to food and water. Animal protocols were approved by the Gothenburg Animal Ethics Committee (Ethic numbers 163-2014 and 58-2016). The whole experimental process is shown in Fig. 1. A total of 270 WT (male 134, female 136), 108 *Tcrd* ^−/−^ (male 51, female 57), and 105 *Tcra* ^−/−^ (male 51, female 54) mice were used in this study.

### LPS administration

The TLR4 ligand LPS (*E. coli* 055:B5, Catalog no. 423, Lot no. 4231A1) was purchased from List Laboratories. Two doses of LPS (5 and 10 mg/kg, in sterile saline) were used to test the induction of white matter injury in WT mice as described in the “Results.” Saline-injected mice served as controls. For induction of sepsis, LPS doses were injected subcutaneously between the shoulders of PND 2 mice as previously described [9].

### Isolation of mouse spleen and brain mononuclear cells and flow cytometry analysis

The neonatal mouse spleens were dissected out after cardiac perfusion with saline at different time points after induction of sepsis with LPS injection. Single cell suspensions were obtained from the neonatal mouse spleen and labeled with the following antibodies: anti-CD45R (FITC, clone RA3-6B2; eBioscience), anti-CD11c (APC-Cy7, clone N418; BioLegend), anti-CD4 (APC, clone GK1.5; eBioscience), and anti-CD8a (PE, clone 53-6.7, eBioscience) for the subsets of conventional dendritic cells (cDCs) or anti-CD3e (FITC, clone 145-2C11; eBioscience), anti-TCRγδ (PE-Cy7, clone GL3; eBioscience), anti-CD11c (PE, clone HL3; BD Biosciences), anti-CD69 (APC, clone H1.2F3; BD Biosciences), and anti-CD86 (BV421, clone GL-1; BD Biosciences) for the detection of γδT cells and DCs as well as the activation status of these cells.

The neonatal mouse brains were dissected out after cardiac perfusion with saline and incubated with an enzyme mixture containing 0.01% papain, 0.01% DNase I (Worthington, NJ, USA), 0.1% Dispase II (Roche, Sweden), and 12.4 mM MgSO_4_ in Ca^+^/Mg^+^-free HBSS (Thermo Fisher, Sweden). The single cell suspensions were obtained through Percoll (30/70%) gradient procedures. The antibodies used were anti-CD45 (APC-Cy7, clone 30-F11; BD Biosciences), anti-CD3e (FITC, clone 145-2C11; eBioscience), anti-TCRγδ (PE-Cy7, clone GL3; BD Biosciences), anti-CD11c (PE, clone HL3; BD Biosciences), and anti-CD69 (APC, clone H1.2F3; BD Biosciences).

After staining, spleen and brain samples were immediately run on a BD FACSCanto II™ flow cytometer. Data were analyzed with FlowJo software (Tree Star, Ashland, OR, USA), and fluorescence minus one (FMO) controls for each fluorochrome were used for accurate gating of positively stained populations.

### Immunochemistry staining and white matter injury evaluation in mice

PND 12, 26, and 60 mice were chosen to examine the white matter injury because these time points represent different myelination stages in rodents, and thus, the extent of white matter injury can be measured by the presence of myelin basic protein (MBP) [19]. The mice were injected with LPS (5 mg/kg) at PND 2 and then sacrificed at PND 12, 26, or 60. After anesthetization with 50 mg/mL pentothal, mice were perfused intracardially with saline followed by 5% buffered formaldehyde (Histofix; Histolab, Gothenburg). Brains were dissected out and cut into 10-μm coronal sections, and every 50th section was used for histological staining as previously described [20, 21]. For immunostaining and corpus callosum subcortical white matter analyses, every 30th section was used. The primary antibody was mouse anti-MBP (SMI 94; Sternberger Monoclonal, Lutherville, MA, USA), and the secondary antibody was horse anti-mouse IgG. The subcortical MBP^+^ white matter volume (mm^3^) of the whole mouse brain or three levels at the corpus callosum of the mouse brain were calculated as previously described [20, 22] using the following formula: *V* = *S*_A_ × *p* × *T*, where *V* is the total volume, *S*_A_ is the sum of the areas measured, *p* is the inverse of the section sampling fraction, and *T* is the section thickness.

To analyze cortical myelination, as an indication of myelinated axons, the length of myelinated fibers within the cortex was measured between the external capsule and the cortical plate at fixed levels and fixed distance from the cingulum. The MBP-positive area in the cortex and the density of MBP-positive staining in the cortex were determined by using ImageJ software and manually setting threshold to include MBP-stained cortical area, followed by measuring the proportion of the field that was positive for MBP staining in the cortex. The MBP immunodensity was determined by measuring integrated density and normalized to the saline group.

### Mouse elevated plus maze

The elevated plus maze is one of the most widely used behavioral tests for measuring anxiety in rodents. The maze was made from black Plexiglas and placed on an aluminum stand 60 cm above the floor. The maze is usually a cross-shaped maze with two open arms (open areas) and two closed arms (closed areas), and mice with more entries into the open arms indicate reduced anxiety [23]. At PND 26, the animals were placed in one closed arm of the maze at the start of the measurement and then recorded individually for 5 min. The time spent in the closed and open arms and the number of entries into the open arm were analyzed manually. The test was performed in the morning to avoid the daily variation of hormones that could interfere with the test, and males and females were tested separately. The room was only dimly illuminated and was kept quiet during the test.

### Mouse gait analysis

Mouse gait data were quantified using the DigiGait Imaging System (Mouse Specifics, Inc.), which was described in detail previously [24]. This test is used for the accurate assessment of treadmill and overground locomotion, as well as the integrity of the cerebellum and muscle tone/equilibrium [25–27]. Briefly, mice are allowed to walk on a motorized transparent treadmill belt with a high-speed video camera mounted below to capture the images of paw prints on the belt. Each paw of the animal was treated as a paw area, and its position was recorded relative to the belt. Animals were tested at PND 26 at a speed of 25 cm/s and at PND 56 at 20 cm/s. An average of ten sequential strides per paw was recorded for each mouse, which is sufficient for the analysis of gait behaviors in mice [28]. Eight parameters were used to show the behavior performance of the mice, and each parameter of the fore and hind paws was the mean value of the left and right fore and hind paws, respectively.

### Statistics

Based on our previous studies on mouse models with prenatal brain injury [29] and our pre-experiment results for behavior tests, a group size with around 15 mice is sufficient to evaluate differences in behavior changes (*p* < 0.05, two-tailed *t*-test) with the power of 0.75. For the flow cytometry analysis of the different cDCs and γδT cell populations, the results represent one of at least three independent experiments. The SPSS statistics software (version 19.0, IBM) was used for data analysis. Quantitative data are presented as means ± SD and were analyzed by one-way ANOVA or two-way ANOVA. The least significant difference (LSD) test was used to compare individual means when appropriate. The limit of statistical significance was *p* < 0.05.

## Results

### Sepsis-induced disruption of white matter development in newborn mice

LPS-induced sepsis is often triggered by cells expressing different TLRs. The cell surface TLRs are specialized in recognizing microbial membrane components, while the intracellular TLRs are specialized in recognizing DNA and double-stranded RNA derived from viruses and bacteria [30]. TLR4 is the receptor for LPS, and it also senses endogenous ligands that act as danger signals, including high-mobility group box 1, hyaluronan, heat shock protein 60, and free fatty acids [31–33]. These represent the most common causative pathogens and potential endogenous danger signals in perinatal brain injury. LPS also activates DCs and T cells. We first analyzed how LPS influences white matter development in newborn mice by treating the mice at PND 2.

**Fig. 2:**
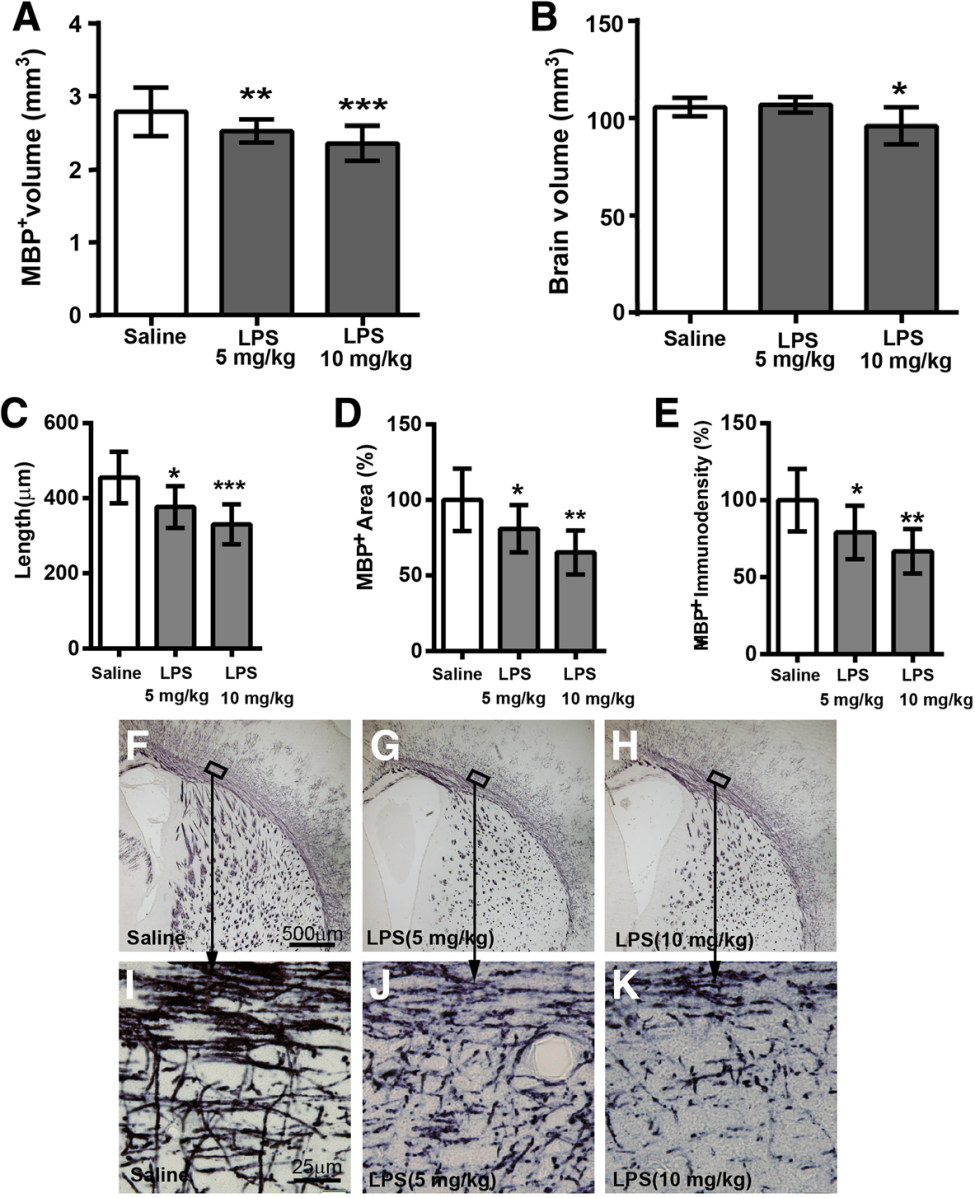
Disruption of white matter development in mice at PND 12 induced by two different doses of LPS at PND 2. **a** White matter volume assessed as a volume of MBP-positive staining. **b** The total brain volume. **c** The length of MBP-positive fibers between the corpus callosum and the end of the fibers in the cortex. **d**, **e** The MBP-positive areas (**d**) and density (**e**) in the cortex, presented as the percentage of saline-treated controls. **f**–**k** The representative photos show MBP-positive staining in saline-treated (**f**, **i**), 5 mg/kg LPS-treated (**g**, **j**), and 10 mg/kg LPS-treated (**h**, **k**) mice. **i**–**k** High-magnification pictures of **f**–**h**, respectively. Saline = 9 mice, LPS (5 mg/kg) = 9 mice, LPS (10 mg/kg) = 8 mice. Data are presented as means ± SD. LPS vs. saline; *p < 0.05, **p < 0.01, ***p<0.001, by one-way ANOVA

We tested two doses of LPS (5 and 10 mg/kg), as shown in Fig. 2, and LPS disrupted white matter development compared to the saline-treated group. Compared to saline-administered control mice at PND 12, the total subcortical white matter volume in the corpus callosum was significantly decreased in LPS-administered mice (Fig. 2a), but no influence on brain volume with the dose of 5 mg/kg (Fig. 2b).

LPS-treated mice also showed significantly shorter myelinated fibers within the cortex (Fig. 2c), and they have a significant reduction of the total positive MBP-stained areas (Fig. 2d) and density (Fig. 2e) in the cortex compared to the control mice. In addition, both doses of LPS-treated mice show fewer, shorter, fragmented, and disorganized myelinated fibers both in the subcortical corpus callosum and in the cortex, compared to the saline-treated mice (Fig. 2f–k), indicating the disruption of the microstructural integrity of myelinated axons.

The 10 mg/kg dose caused more obvious myelin disruption (Fig. 2a, c–e, h, k) compared to the 5 mg/kg dose (Fig. 2a, c–e, g, j) and to saline (Fig. 2a, c–e, f, i), but it also caused a reduction in brain size (Fig. 2b, *p* < 0.01) and caused high mortality (35%) in the treated mice. Thus, 5 mg/kg of LPS was used in all further studies.

### Loss of γδT cells or αβT cells alone failed to affect normal white matter development

**Fig. 3:**
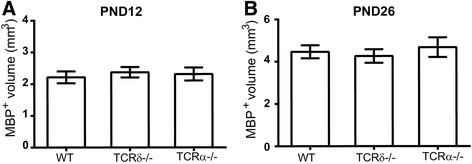
Loss of TCRαβ or TCRγδ does not affect white matter development at different developmental stages in naive mice. White matter volume at PND 12 (**a**) and PND 26 (**b**) in naive WT, *Tcrd* ^−/−^, and *Tcra* ^−/−^ mice. PND 12: WT = 6 mice, *Tcrd* ^−/−^ = 6 mice, *Tcrα* ^−/−^ = 6 mice; PND 26: WT = 15 mice, *Tcrd* ^−/−^ = 12 mice, *Tcrα* ^−/−^ = 14 mice, using one-way ANOVA

LPS induces DC changes that subsequently regulate the T cell-mediated responses [18, 34, 35], and these responses might contribute to the sepsis-induced disruption of white matter development in mice. To define which T cell subsets play an essential role in this process, we used *Tcrd* ^−/−^ and *Tcrα* ^−/−^ mice, which lack γδT cells and αβT cells, respectively. In mice without LPS treatment, we observed that normal white matter development was not affected in either the *Tcrd* ^−/−^ or *Tcra* ^−/−^ mice compared with WT normal control mice at different development stages (Fig. 3a, b).

### γδT cells but not αβT cells contributed to the sepsis-induced disruption of white matter development

**Fig. 4:**
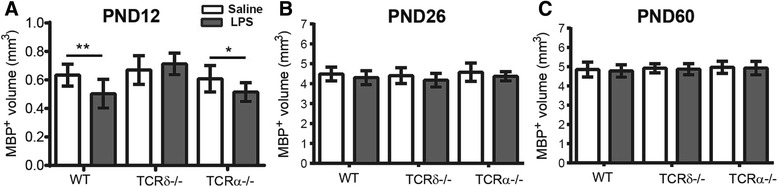
After LPS-induced sepsis, different TCRs affect the development of white matter differently at PNDs 12, 26, and 60. **a** White matter volume of the corpus callosum at PND 12 after LPS injection in all three genotypes (WT: saline = 7 mice, LPS = 8 mice; *Tcrd* ^−/−^: saline = 7 mice, LPS = 9 mice; *Tcrα* ^−/−^: saline = 5 mice, LPS = 6 mice). **b** White matter volume at PND 26 after LPS injection in all three genotypes (WT: saline = 16 mice, LPS = 23 mice; *Tcrd* ^−/−^: saline = 18 mice, LPS = 19 mice; *Tcrα* ^−/−^: saline = 16 mice, LPS = 15 mice). **c** White matter volume at PND 60 after LPS injection in all three genotypes (WT: saline = 24 mice, LPS = 24 mice; *Tcrd* ^−/−^: saline = 16 mice, LPS = 21 mice; *Tcrα* ^−/−^: saline = 18 mice, LPS = 21 mice). Data are presented as means ± SD; two-way ANOVA with LSD for pairwise comparisons, *p < 0.05, **p < 0.01

We then analyzed the white matter development in different mouse strains treated with LPS. WT, *Tcrd* ^−/−^, and *Tcra* ^−/−^ mice were subcutaneously injected with saline or 5 mg/kg LPS at PND 2, and white matter was evaluated at different developmental stages (PNDs 12, 26, and 60). At the early stage of brain development after sepsis (PND 12), the WT mice treated with LPS displayed myelin development disruption (Fig. 4a). Interestingly, *Tcrα* ^−/−^ mice displayed a similar myelin deficiency at PND 12 as was seen in the WT mice, but myelin disruption was not observed in *Tcrd* ^−/−^ mice after the same treatment (Fig. 4a, *F*_2, 41_ = 11.248, *p* < 0.0001, main effect of genotype; *F*_1, 41_ = 5.731, *p* = 0.021, main effect of treatment by two-way ANOVA).

At a later stage of brain development after LPS administration (PND 26), the disruption in white matter development had recovered in both WT mice and *Tcrα* ^−/−^ mice (Fig. 4b, *F*_2, 101_ = 2.25, *p* = 0.111, main effect of genotype; *F*_2, 101_ = 8.437, *p* = 0.005, main effect of treatment by two-way ANOVA). By PND 60, white matter development had recovered in all three genotypes of mice examined (Fig. 4c, *F*_2, 118_ = 1.827, *p* = 0.165, main effect of genotype; *F*_1, 118_ = 0.819, *p* = 0.367, main effect of treatment by two-way ANOVA). Taken together, the lack of γδT cells, but not the lack of αβT cells, rescued the transient myelin development disruption caused by sepsis in early life.

### Sepsis caused changes in cDC subsets

LPS can induce the activation/maturation of cDCs and then can activate γδT cells [18, 34, 35]; therefore, we next examined the presence of different cDC populations and their maturation and activation status in the periphery and brain. The cDCs comprise CD4^−^CD8a^−^, CD4^−^CD8a^+^, CD4^+^CD8a^−^, and CD4^+^CD8a^+^ subsets, with CD4^−^CD8a^+^ cDCs being the most mature cDC subset. In contrast to adult mice, neonatal mice in early life have an immature and Th2-biased immune system partly due to the low percentage of the CD4^−^CD8a^+^ mature cDC subset [36, 37].

**Fig. 5:**
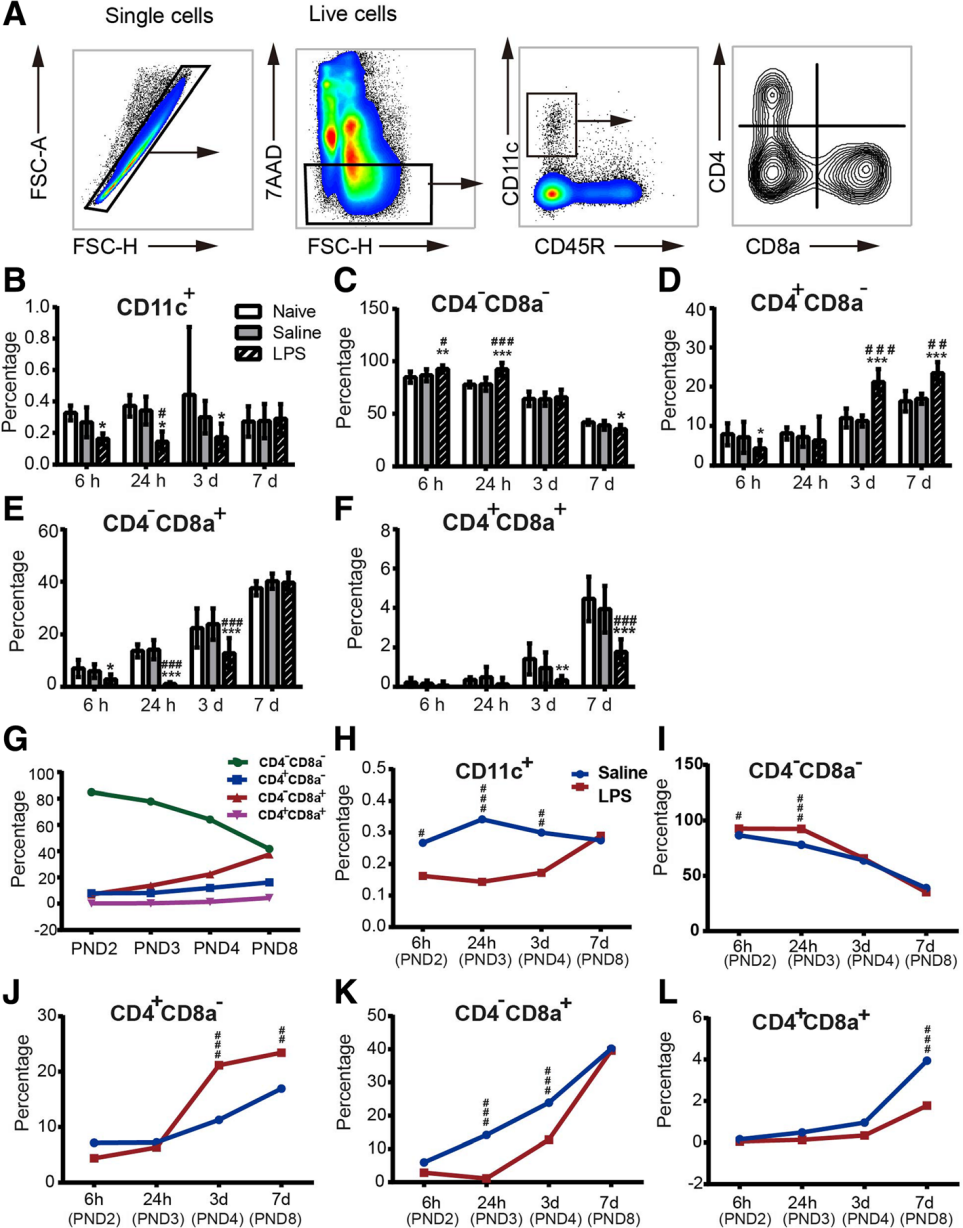
The dendritic cells in the spleen after LPS-induced sepsis in mice. **a** The gating strategies for analyzing the dendritic cells and dendritic cell subsets. **b** The percentage of CD11c^+^ cells among all live splenocytes at 6 h, 24 h, 3 days, and 7 days in naive, saline-treated, and LPS-treated mice. **c**–**f** The percentage of different dendritic cell subsets among the total cDCs (CD11c^+^) in the mouse spleen. **g** The percentage of different cDC subpopulations among the total live splenocytes in the normal WT mice. **h**–**l** The percentage of total cDCs among the total live splenocytes (**h**) and different subpopulations of cDCs among the total cDCs (**i**–**l**) in the WT mouse spleen after LPS treatment. At 6 h: naive = 6 mice, saline = 7 mice, LPS = 12 mice; at 24 h: naive = 6 mice, saline = 5 mice, LPS = 7 mice; at 3 days: naive = 5 mice, saline = 6 mice, LPS = 7 mice; at 7 days: naive = 5 mice, saline = 6 mice, LPS = 7 mice. Data are presented as means ± SD; asterisk, LPS vs. naive; hashtag, LPS vs. saline; *p < 0.05, **p < 0.01, ***p < 0.001, ^#^ *p* < 0.05, ^##^ *p* < 0.01, ^###^ *p* < 0.001, by two-way ANOVA with LSD for pairwise comparisons

Because LPS-induced sepsis is a systemic disease, we first analyzed the impact of LPS-induced sepsis on the peripheral cDC subsets of neonatal mice. We used flow cytometry to determine the total number of cDCs (CD45R^−^CD11c^+^) and different cDC subsets in the WT mouse spleen at the different developmental stages (Fig. 5a–l). During normal development, the percentage of total cDCs among the total live splenocytes did not change as the mouse developed and remained around 0.30% (Fig. 5b, naive group). However, the percentage of immature cDCs (CD4^−^CD8a^−^) among the total cDCs decreased from about 85% at PND 2 to about 41% at PND 8 (Fig. 5c), and this subpopulation changed the most during development (Fig. 5g). In contrast, the percentage of mature cDCs (CD4^−^CD8a^+^) increased with development from 7% at PND 2 to 37% at PND 8 (Fig. 5e, g).

LPS administration at PND 2 caused a decrease in total cDCs in the spleen at 6 h, 24 h, and 3 days after LPS compared to the saline group (*p* < 0.05, Fig. 5b, h). The immature cDCs (CD4^−^CD8a^−^) first increased at 6 and 24 h and then decreased at 7 days in the spleen after LPS injection (*p* < 0.05, Fig. 5c, i), while the CD4^+^CD8a^−^ cells initially decreased at 6 h but increased at 3 and 7 days after LPS injection (*p* < 0.05, Fig. 5d, j). The CD4^−^CD8a^+^ cells (mature cDCs) also decreased at 6 h, 24 h, and 3 days after LPS injection but returned to baseline at 7 days (*p* < 0.05, Fig. 5e, k), and the CD4^+^CD8a^+^ cells decreased during the first week after LPS injection (*p* < 0.05, Fig. 5e, l).

### Sepsis caused increased γδT cell activation in the spleen but not in the brain

We next analyzed the sepsis-induced changes in DCs and γδT cells in the brain. Our recent publication showed that in comparison to the TLR2 agonist Pam3CSK4, LPS stimulation induces significantly lower numbers of brain-infiltrating monocytes and neutrophil cells at 14 and 48 h [38]. Therefore, we analyzed the presence and activation status of DCs and γδT cells in the brain and the spleen at these two time points.

**Fig. 6:**
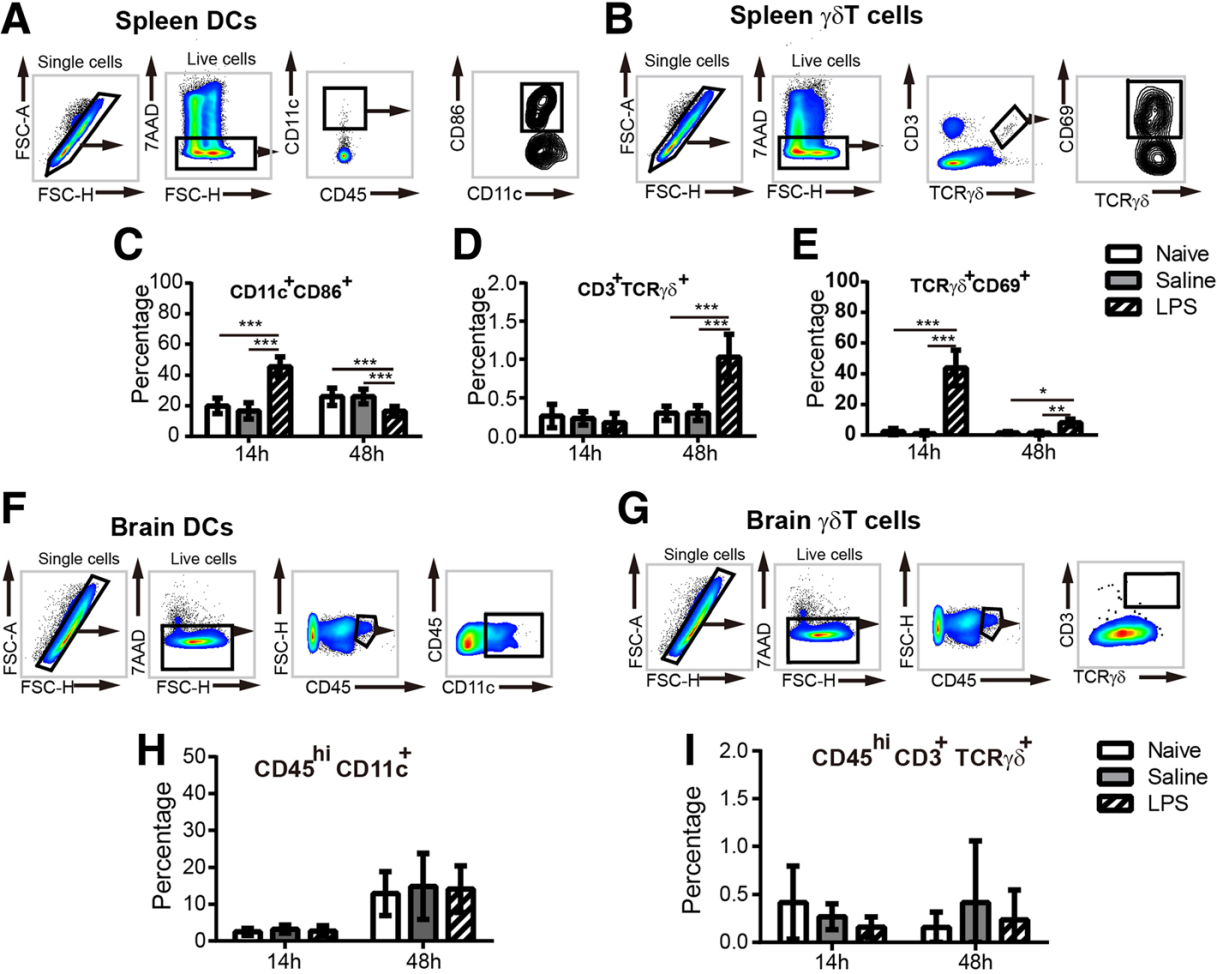
The presence and activation of dendritic cells and γδT lymphocytes in the spleen and brain after LPS-induced sepsis. **a** The gating strategies for analyzing CD11c^+^ dendritic cells and CD86 expression in the spleen. **b** The gating strategies for analyzing γδT lymphocytes and CD69 expression in the spleen. The percentage of CD11c^+^CD86^+^ cells (**c**) in the spleen at 14 and 48 h after LPS injection. The percentage of CD3^+^TCRγδ^+^ cells (**d**) and CD3^+^TCRγδ^+^CD69^+^ cells (**e**) in the spleen at 14 and 48 h after LPS injection. At 14 h: naive = 6 mice, saline = 6 mice, LPS = 6 mice; at 48 h: naive = 5 mice, saline = 7 mice, LPS = 9 mice. **f** The gating strategies for analyzing the dendritic cells in the brain. **g** The percentage of CD45^hi^CD11c^+^ cell populations in the brain at 14 and 48 h after LPS injection. **h** The gating strategies for analyzing the γδT lymphocytes in the brain. **i** The percentage of CD45^hi^CD3^+^TCRγδ^+^ cells in the brain at 14 and 48 h after LPS injection. At 14 h: naive = 6 mice, saline = 3 mice, LPS = 9 mice; at 48 h: naive = 5 mice, saline = 5 mice, LPS = 6 mice. All data are presented as means ± SD. *p < 0.05, **p < 0.01, ***p < 0.001, by two-way ANOVA with LSD for pairwise comparisons

The frequencies of activated (CD86^+^CD11c^+^) DCs were increased in the spleen at 14 h after LPS administration, suggesting that LPS activated splenic DCs (Fig. 6a, c). We also observed that LPS increased the percentage of splenic γδT cells at 48 h and activated these cells at both 14 and 48 h after injection, as judged by CD69 expression on these γδT cells (Fig. 6b, d, e). Although there were DCs and a few γδT cells present in the brain, we did not observed increased infiltration of these cells into the brain at either time point in LPS-treated mice compared to saline controls (Fig. 6f–i).

### Neither sepsis nor γδT cell or αβT cell deficiency alone had an impact on anxiety-related behaviors in mice

Infection during early life not only affects brain development, but it also might have subsequent neurobehavioral impacts later in life. To evaluate such a possible effect, we performed behavior tests after LPS treatment in WT, *Tcrd* ^−/−^, and *Tcra* ^−/−^ mice.

**Fig. 7:**
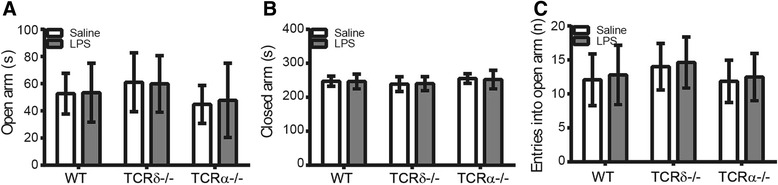
No anxiety-related behaviors were found in WT, *Tcrd* ^−/−^, or *Tcra* ^−/−^ mice at PND 26. **a** Time in the open arm of the elevated plus maze. **b** Time in the closed arm. **c** The number of entries into the open arm. WT: saline = 27 mice, LPS = 24 mice; *Tcrd* ^−/−^: saline = 14 mice, LPS = 21 mice; *Tcra* ^−/−^: saline = 20 mice, LPS = 23 mice. Data are presented as means ± SD; two-way ANOVA with LSD for pairwise comparisons

We tested for anxiety-related behaviors in mice using the elevated plus maze at PND 26 and found that LPS-induced sepsis did not lead to anxiety-related behaviors in WT mice later in life, nor did the *Tcrd* ^−/−^ or *Tcrα* ^−/−^ genotypes (Fig. 7). All of the LPS-treated mice spent similar times in the open arm of the elevated plus maze compared with each of their corresponding saline-treated controls (Fig. 7a, *F*_1, 123_ = 0.049, *p* = 0.826, main effect of treatment by two-way ANOVA), and the *Tcrd* ^−/−^ and *Tcrα* ^−/−^ mice also spent similar times in the open arm compared with WT controls (Fig. 7a, *F*_2, 123_ = 4.567, *p* = 0.012, main effect of genotype by two-way ANOVA). There were also no differences between saline and LPS groups of the three genotypes in terms of time spent in the closed arm (Fig. 7b, *F*_2, 123_ = 0.049, *p* = 0.826, main effect of genotype; *F*_1, 123_ = 4.567, *p* = 0.012, main effect of treatment by two-way ANOVA). Similarly, neither LPS administration nor genotype had any effect on the number of entries into the open arm (Fig. 7c, *F*_2, 123_ = 0.959, *p* = 0.329, main effect of genotype; *F*_1, 123_ = 3.635, *p* = 0.029, main effect of treatment by two-way ANOVA).

### Transient motor function abnormality in LPS-induced sepsis was dependent on γδT cells but not αβT cells

We also explored the influence of sepsis, as well as T cell deficiency, on motor function using treadmill-based DigiGait analysis, which is a powerful method for detecting subtle changes in posture and locomotion compared to many of the other common behavior tests [27]. A total of eight gait parameters were observed, including swing duration, propulsion duration, stance duration, stride duration, stride length, stance width, stride frequency, and step angles. The parameters and a representative image for the test are shown in Additional file 1A–D.

**Fig. 8:**
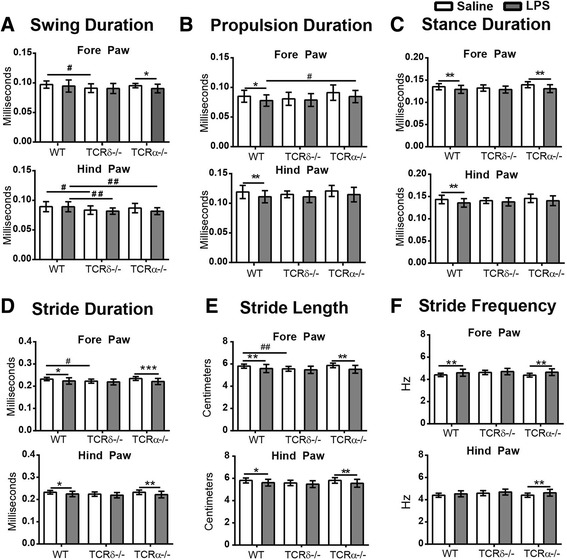
Gait profile in WT, *Tcrd* ^−/−^, and *Tcra* ^−/−^ mice at PND 26. Displayed are swing duration (**a**), propulsion duration (**b**), stance duration (**c**), stride duration (**d**), stride length (**e**), stride frequency (**f**), WT: saline = 27 mice, LPS = 22 mice; *Tcrd* ^−/−^: saline = 15 mice, LPS = 22 mice; *Tcrα* ^−/−^: saline = 20 mice, LPS = 20 mice. Data are presented as means ± SD; two-way ANOVA with LSD for pairwise comparisons. Asterisk, saline vs. LPS; *p < 0.05, **p < 0.01, ***p < 0.001. Hashtag, WT vs. *Tcrd* ^−/−^ or WT vs. *Tcrα* ^−/−^; ^#^ *p* < 0.05, ^##^ *p* < 0.01

LPS-induced sepsis at PND 2 caused motor abnormalities in WT mice at PND 26 (Fig. 8), including decreased durations of swing, propulsion, stance, and stride as well as decreased stride lengths for both the fore paw and hind paw (Fig. 8a–e). The stride frequency of the fore paw in the LPS-treated WT mice was significantly higher than that in the saline-treated mice (Fig. 8f), while the stance width and step angle were not influenced by early-life sepsis in WT mice (data not shown). No significant differences between males and females were observed (data not shown).

Similarly, at PND 26, the LPS-treated *Tcra* ^−/−^ mice displayed motor abnormalities (Fig. 8) in comparison to the saline control groups, including decreased durations of swing (fore paw), stance (fore paw), and stride (both paws) (Fig. 8a–d). These mice also had decreased stride length for both paws (Fig. 8e) and increased stride frequency (both paws, Fig. 8f).

In contrast, at PND 26, in the LPS-treated *Tcrd* ^−/−^ mice, there were no significant differences in any of the parameters observed in comparison to the saline-treated control *Tcrd* ^−/−^ mice (Fig. 8). A lack of sepsis-induced motor function impairment in *Tcrd* ^−/−^ mice suggests that the lack of γδT cells might also rescue the sepsis-induced disruptions in brain motor functional development.

Interestingly, deficiency in αβT cells in *Tcra* ^−/−^ mice had an impact on motor function because, compared to WT mice, saline-administrated control *Tcrα* ^−/−^ mice had a significantly shorter swing duration (fore paw, Fig. 8a), stride duration (fore and hind paws, Fig. 8d), and stride length (fore and hind paws, Fig. 8e).

To further explore the influence of sepsis and the role of T cells on neurobehavioral development, we tested motor function again using DigiGait analysis at adult age (PND 56) in WT, *Tcrα* ^−/−^, and *Tcrd* ^−/−^ mice. The abnormalities in most gait parameters observed at PND 26 had recovered at PND 56 except for the observation that the propulsion duration of the hind paw was still significantly shorter in LPS-administered *Tcrα* ^−/−^ mice compared to saline-administered *Tcrα* ^−/−^ control mice (*F*_1, 103_ = 4.32, *p* = 0.04, main effect of treatment by two-way ANOVA) (data not shown). However, we did not observe any significant sex-dependent differences in white matter injury, anxiety, and gait behavior in all of three mouse strains.

## Discussion

In infants, the immune system and CNS continue to develop after birth, and clinical and animal studies have shown that communication between the immune system and the CNS influences brain development, function, and behavior. Here, we report that LPS-induced sepsis in mice induces disruption/injury to the white matter in the brain and that this is dependent on γδT cells, but not αβT cells. To our knowledge, this is the first study showing the important role of γδT cells in LPS-induced preterm brain injury.

Both γδT cells and αβT cells have been shown to be important in CNS pathologies [39] such as multiple sclerosis [40] and stroke [41]. In contrast to αβT cells that require antigen processing and presentation on MHC molecules, γδT cells do not strictly require antigen presentation and act more as innate-like cells [42]. During development, the γδTCRs are the first to be expressed on murine [43, 44] and human [45] thymocytes, and αβT cells arise later and appear to be immature during the infancy period [46–48]. The evolutionary nature of γδT cells points out their critical role in the regulation of immune responses in early life. Indeed, using a neonatal rodent model of LPS-induced sepsis, we have shown here that sepsis-induced white matter disruption and injury, as well as motor function abnormality, require the presence of γδT cells but not αβT cells. The relatively early occurrence of γδT cells in neonates compared to αβT cells might also explain the contrasting results regarding the role of γδT cells and αβT cells in LPS-induced sepsis in adult mice from other studies [49–51]. Therefore, our findings have provided clear evidence that γδT cells are the critical T cell subsets that promote the LPS-induced preterm brain injury in neonatal mice.

The critical role of γδT cells in LPS-induced preterm brain injury does not seem to depend on increased numbers of γδT cells in the mouse brain because LPS administration did not induce increased γδT cell infiltration as evaluated by flow cytometry analyses of brain-infiltrating leukocytes, and this agrees with our recent publication that LPS does not significantly cause immune cell infiltration into the CNS when compared to the TLR2 agonist Pam3CSK4 [38]. Instead, our results suggest that LPS might activate γδT cells both directly and indirectly through activation of cDCs in the periphery (e.g., the spleen), leading to γδT cell proliferation, and consequently increased γδT cells in the periphery later in life. Accordingly, LPS caused an increase in the frequency of CD8a^−^ immature cDCs but a decrease in mature CD8a^+^ cDCs in the spleen. It is likely that LPS induced proliferation of immature cDCs and the activation and egress of mature cDCs from the spleen that, in turn, activated γδT cells, as reported previously [18, 34, 35]. This caveat warrants further investigation into how cDCs contribute to the immune response and CNS injury in newborns by modulating γδT cells and/or other immune cells, as well as investigation into the effector mechanisms through which γδT cells promote sepsis-induced preterm brain injury, which were not addressed in the current study.

Mice deficient in both γδT cells and αβT cells (*Tcrd* and *Tcra* double-knockout mice) display impaired CNS circuitry and increased anxiety-like behavior [52], suggesting that T cells regulate the development of exploratory and anxiety-related behaviors. In the present study, we analyzed the separate effects of deficiency in γδT cells and αβT cells on brain white matter injury and neurobehavior in neonatal mice. Although deficiencies in γδT cells or αβT cells alone failed to impact normal white matter development, genetic deletion of γδT cells, but not αβT cells, rescued the LPS-induced transient white matter disruption and thus rescued the motor function abnormalities. This further suggests that γδT cells, but not αβT cells, might contribute to the early stage of white matter injury as well as to transient motor function abnormalities later in life in sepsis-induced preterm mice.

γδT cells not only promoted sepsis-induced white matter injury but also caused a defect in motor function in subsequent later life. This new finding has for the first time indicated that γδT cells can cause neurobehavior abnormalities in later life, which might contribute to the development of cerebral palsy. In this study, we used a very sensitive method aimed at detecting subtle changes on motor function because infants with subtle perinatal brain injury might develop neurodevelopmental abnormalities [53]. Indeed, we observed significant gait abnormality in both WT and *Tcrα* ^−/−^ mice but not in *Tcrd* ^−/−^ mice after LPS treatment, consistent with our finding that genetic deletion of γδT cells rescued the sepsis-induced white matter injury. Early detection of subtle changes in motor function using a sensitive method in human infants might thus be very meaningful and clinically significant in that it may expedite the rehabilitation process for the affected children. In fact, studies in preterm infants with white matter disruption and children with cerebral palsy [54–56], as well as in adult patients with white matter injury [57, 58], have shown that they all display subtle changes in gait and the coordination of leg movements. In contrast, we failed to observe any effect on anxiety behavior in adult mice (PND 56), independent of mouse genotype. It is possible that LPS-induced stress might change the composition of immune cells or the immune response, and thus, our different observations compared to those reported in the literature are likely to be experiment- and context-dependent [59, 60].

## Conclusions

In summary, our findings suggest that the sepsis-induced disruption of white matter development in mice in early life and transient motor function at older age requires γδT cells but not αβT cells. Such a critical role of γδT cells does not appear to depend on increased numbers of γδT cells in the mouse brain but might require LPS-induced proliferation of immature cDCs, activation and egress of mature cDCs from the periphery, and subsequent activation of γδT cells. This finding provides important evidence for the role of γδT cells in the interactions between the immune system and the CNS in early life and suggests new therapeutic strategies against sepsis-induced perinatal brain injury.

## Additional file

Additional file 1: Treadmill-based measurement of gait properties. (A) A representative image from the video recording of a mouse running on the treadmill at 25 cm/s at PND26. (B) Digital print of each paw illustrated in (A). (C) Graph of paw area in contact with the treadmill surface over time for a representative single paw showing stride duration, swing duration, and stance duration. (D) The definitions for stance width, stride length, and step angle. (JPEG 227 kb)

## Abbreviations

CD: Cluster of differentiation
cDCs: Conventional dendritic cells
DCs: Dendritic cells
DigiGait: DigiGait imaging system
LPS: Lipopolysaccharide
MBP: Myelin basic protein
MHC: Major histocompatibility complex
PND: Postnatal day
TCR: T cell receptor
*Tcrd*^−/−^: TCRδ deficient
*Tcrα*^−/−^: TCRα deficient
TLR: Toll-like receptor
WT: Wild type
