# Supplementary figures and images for: γδT cells but not αβT cells contribute to sepsis-induced white matter injury and motor abnormalities in mice
Source: J Neuroinflammation. 2017 Dec 20;14:255. doi: 10.1186/s12974-017-1029-9 (PMC5738716; doi:10.1186/s12974-017-1029-9)

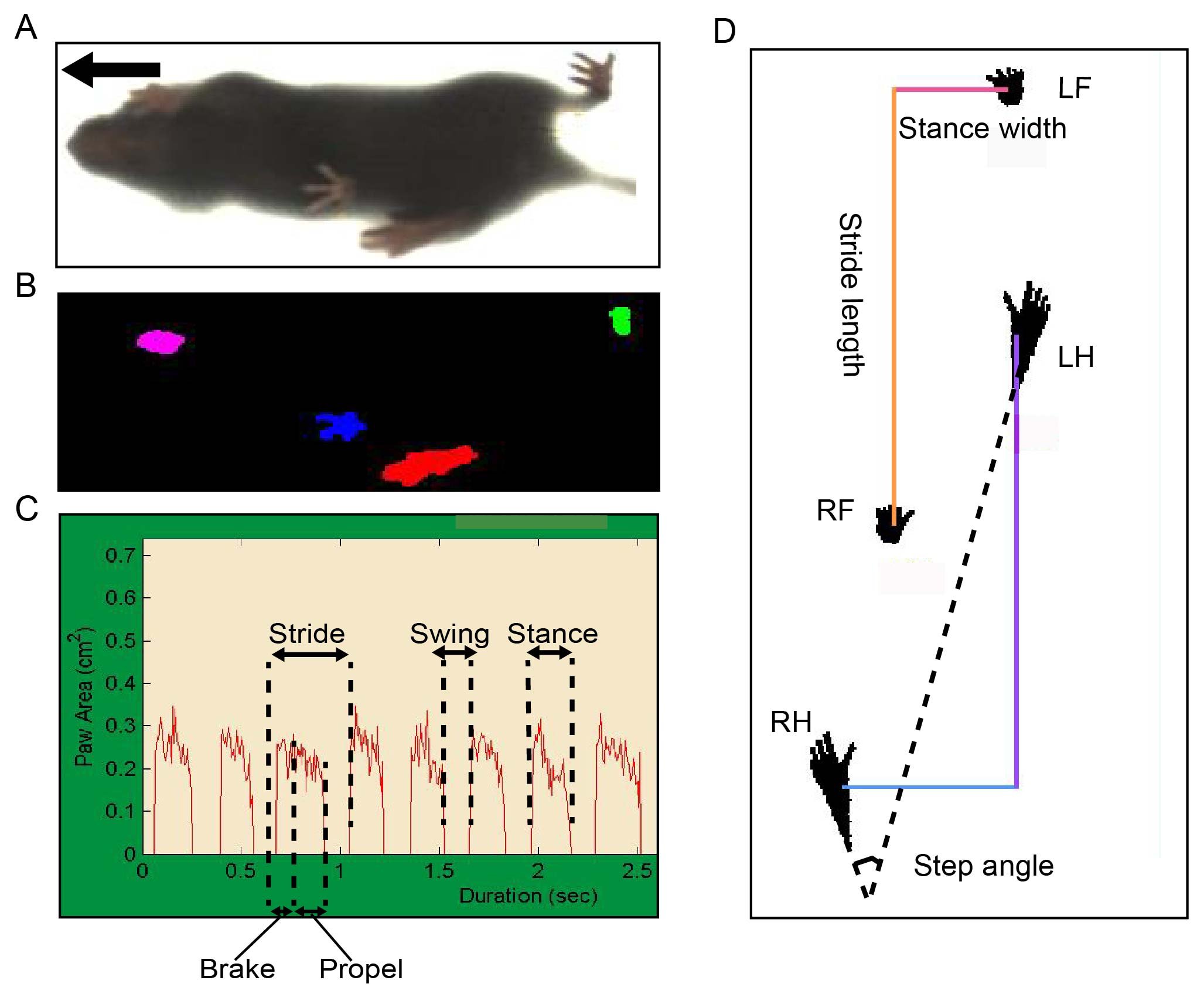

Supplement: Additional file 1: — Treadmill-based measurement of gait properties. (A) A representative image from the video recording of a mouse running on the treadmill at 25 cm/s at PND26. (B) Digital print of each paw illustrated in (A). (C) Graph of paw area in contact with the treadmill surface over time for a representative single paw showing stride duration, swing duration, and stance duration. (D) The definitions for stance width, stride length, and step angle. (JPEG 227 kb) [file 12974_2017_1029_MOESM1_ESM.jpg]
